# The Use of Blockchain Technology in the Health Care Sector: Systematic Review

**DOI:** 10.2196/17278

**Published:** 2022-01-20

**Authors:** Deepa Elangovan, Chiau Soon Long, Faizah Safina Bakrin, Ching Siang Tan, Khang Wen Goh, Siang Fei Yeoh, Mei Jun Loy, Zahid Hussain, Kah Seng Lee, Azam Che Idris, Long Chiau Ming

**Affiliations:** 1 School of Pharmacy KPJ Healthcare University College Nilai Malaysia; 2 Faculty of Computing and Engineering Quest International University Ipoh Malaysia; 3 Faculty of Information Technology INTI International University Nilai Malaysia; 4 Department of Pharmacy National University Hospital Singapore Singapore; 5 Faculty of Engineering Universiti Teknologi Malaysia Skudai Malaysia; 6 Faculty of Health University of Canberra Canberra Australia; 7 Faculty of Pharmacy University of Cyberjaya Cyberjaya Malaysia; 8 Faculty of Integrated Technologies Universiti Brunei Darussalam Gadong Brunei Darussalam; 9 Pengiran Anak Puteri Rashidah Sa’adatul Bolkiah Institute of Health Sciences Universiti Brunei Darussalam Gadong Brunei Darussalam

**Keywords:** blockchain, health care, hospital information system, data integrity, access control, data logging, health informatics

## Abstract

**Background:**

Blockchain technology is a part of Industry 4.0’s new Internet of Things applications: decentralized systems, distributed ledgers, and immutable and cryptographically secure technology. This technology entails a series of transaction lists with identical copies shared and retained by different groups or parties. One field where blockchain technology has tremendous potential is health care, due to the more patient-centric approach to the health care system as well as blockchain’s ability to connect disparate systems and increase the accuracy of electronic health records.

**Objective:**

The aim of this study was to systematically review studies on the use of blockchain technology in health care and to analyze the characteristics of the studies that have implemented blockchain technology.

**Methods:**

This study used a systematic review methodology to find literature related to the implementation aspect of blockchain technology in health care. Relevant papers were searched for using PubMed, SpringerLink, IEEE Xplore, Embase, Scopus, and *EBSCOhost*. A quality assessment of literature was performed on the 22 selected papers by assessing their trustworthiness and relevance.

**Results:**

After full screening, 22 papers were included. A table of evidence was constructed, and the results of the selected papers were interpreted. The results of scoring for measuring the quality of the publications were obtained and interpreted. Out of 22 papers, a total of 3 (14%) high-quality papers, 9 (41%) moderate-quality papers, and 10 (45%) low-quality papers were identified.

**Conclusions:**

Blockchain technology was found to be useful in real health care environments, including for the management of electronic medical records, biomedical research and education, remote patient monitoring, pharmaceutical supply chains, health insurance claims, health data analytics, and other potential areas. The main reasons for the implementation of blockchain technology in the health care sector were identified as data integrity, access control, data logging, data versioning, and nonrepudiation. The findings could help the scientific community to understand the implementation aspect of blockchain technology. The results from this study help in recognizing the accessibility and use of blockchain technology in the health care sector.

## Introduction

Health informatics (HI) is an extension of medical informatics that concentrates on the clinical sector and implementation of technology in the distribution of health care [1]. Changes in both technology and health care are leading to the evolution of health care informatics. With current technology, HI provides fundamental, indivisible knowledge bases to health professionals and health care organizations to provide patients with a better quality of care services [2]. Acquiring and recording medical and patient information, liaising with health care professionals, choosing an appropriate diagnostic method, elucidating laboratory findings, and gathering clinical research information are known as information processing and communication in the health care sector [3].

Electronic health record (EHR) systems and hospital information systems (HISs) are widely used across the world. However, the current HISs are mainly cloud based, are stored by one particular data contractor, and have several disadvantages, such as a lack of sufficient security measures. This has led to innumerable breaches of data, as well as issues of data validity and data sharing, which have left patients exposed to economic threats and possible social stigma. Centralized data or information is an appealing target for cyberattacks, and issues arise due to establishing a persistent view of the patient data across a network [4].

Taking these issues into consideration, an improved tamperproof and hackproof database management system is much needed to replace the current system that has been used for the past several decades. The new innovative system should have better data security and be able to integrate with other information technology (IT) systems, such as finance and admission systems. When blockchain technology was introduced in 2008, it largely fulfilled all of these criteria, alongside its versatility for applications in banking and finance. 

Blockchain is a decentralized database that maintains an uninterrupted, growing list of data records that are established by the nodes involved. The information is recorded in a public ledger that includes data from every completed transaction [5]. Along with this, blockchain is also a sort of dispersed ledger of cryptographically chained blocks where value-exchanged transactions are consecutively aggregated. Blockchain also exhibits properties such as decentralization, security, anonymity, and data integrity with the absence of a mediator to control agreement and inalterability [6]. The information in blockchain is transparent and tamperproof due to the continuous series of blocks, which contain information and data [7].

Blockchain is a decentralized database that is not owned by anybody and is simultaneously owned by everybody, as the contents are available to all parties involved. For example, with Bitcoin, since all transactions are processed by users via a particular pseudonym, the information contained in the blockchain is completely anonymous [8]. This revolutionary system has indeed overcome some of the limitations faced by the existing system. Nevertheless, further exploration in terms of implementation and practicality is much needed and will be discussed in the following sections [9].

This study aimed to systematically review studies on the use of blockchain technology in health care. We also analyzed the characteristics of the studies that have implemented blockchain technology. This study will be impactful in helping the scientific community to understand the use aspect of blockchain technology based on the findings of completed studies. The results from this study will help to identify the accessibility as well as the implementation of blockchain technology in the health care sector.

## Methods

### Study Design

The methodology used in this research was a systematic review, modeled on a recent systematic review about blockchain reported by Böhme et al [7].

### Data Sources and Search Strategy

A search was conducted for scientific papers on the research topics. All papers that were relevant for these topics were gathered by using a search protocol that was developed for each scientific database. Possible keywords were tested, and appropriate terms were chosen for the search string. The Medical Subject Headings (MeSH) database was used to derive keywords and search term combinations. PubMed, SpringerLink, IEEE Xplore, Scopus, Embase, and *EBSCOhost* databases were chosen to search for all the relevant literature. The search strings were constructed in accordance with the research domains and research questions and are listed in Multimedia Appendix 1.

Online digital libraries were used to search for relevant papers from January 2008 to September 2019. The year 2008 was chosen as the beginning of the range for this research study because the first published application of blockchain technology (ie, Bitcoin) was introduced in that year, so no blockchain-related studies were conducted before 2008. In this systematic review, the search query was purposely made broad, in order to identify many papers related to the research question. However, when “Bitcoin” was used as a search term, a large number of papers were identified, but the papers were mainly about economic applications rather than applications in the health care sector.

Because the aim of this research was based on finding and mapping the papers related to blockchain technology in the health care sector, “Bitcoin” was dropped as a search term. By using “blockchain” and “health care” as search terms, the majority of Bitcoin-related papers with a technical perspective on blockchain were still included. A manual search was carried out for papers that were published at workshops, at conferences, in journals, and at symposiums.

### Study Selection

#### Screening of Relevant Papers

The next step in the process was screening relevant papers, wherein the papers that had been found during the previous step were assessed for actual relevance. The screening process started with all of the publications gathered from online digital libraries. A process inspired by Yli-Huumo et al [10] was used to screen for relevant papers. Applicable quotes from the search were entered into, and sorted with the aid of, EndNote X8.0 (Clarivate), which was used to remove duplicate papers. Duplicate references across databases and references that were not appropriate for the study were eliminated from the literature search reference lists. The remaining duplicates were deleted manually.

The iterative approach for title, abstract, and full-text searches was used, and the results were exported to Microsoft Excel 2013. The titles and abstracts of the searched papers were screened first to determine the relevance or appropriateness for this systematic review. At this stage, studies that were clearly not about the use of blockchain technology in health care were excluded. The titles and abstracts were screened by two reviewers based on the inclusion and exclusion criteria.

#### Inclusion and Exclusion Criteria

The papers that had passed the previous screening phase were screened based on their abstract. In addition, the following specific inclusion and exclusion criteria were used to screen each paper:

1. Inclusion criteria:
  - Original research study.
  - Study in English.
  - Publication on blockchain technology in the health care sector.
  - Publication including sufficient explanation of the research findings.
2. Exclusion criteria:
  - Papers without full-text availability.
  - Papers for which English was not the main language.
  - Papers that had some other focus instead of the use of blockchain in the health care sector.
  - Papers that were duplicates.
  - Search results that were editorials, prefaces, paper summaries, summaries of tutorials, interviews, news items, correspondences, discussions, comments, readers’ letters, workshops, panels, and poster sessions.
  - Publications indicating ideas, magazine publications, and discussion papers.

#### Abstract Screening Based on Keywords

Screening based on keywords, as defined by Dyba and Dingsøyr [11], was done in two steps. In the first step, identifiable keywords and concepts from the abstracts were analyzed that reflected the contribution of the papers. Developing a greater level of understanding based on these keywords was the second step in this keywording process.

The keywords were used to cluster and form categories. All the selected papers were read after the categories had been clustered. The categories were updated after reading each paper, or if the paper revealed something new, then a new category was created. This step resulted in clustered categories being formed from all the relevant papers based on this research topic. Papers with poor, misleading, or lost abstracts were excluded due to irrelevant information.

After the title-, abstract-, and keyword-screening process, each remaining paper underwent full-text screening based on the same eligibility criteria. Two reviewers resolved discrepancies through discussion, and no adjudication by a third reviewer was required.

### Data Gathering and Data Extraction

A template was designed to collect the information required to address the research question. Basic metadata about the publication were collected, such as author name and country, year of publication, source type, and type of publisher.

To categorize the 22 selected papers, further data were extracted. Each full paper was read to extract the keywords or outcomes related to our research question; these were then sorted into the identified categories, as follows:

1. Use cases of blockchain technology in health care that indicate the specific health care area, such as electronic medical records (EMRs), biomedical research and education, remote patient monitoring, drug or pharmaceutical supply chains, health insurance claims, health data analytics, or other areas.
2. Reasons for using blockchain technology in health care, such as data integrity, access control, logging, data versioning, and nonrepudiation.

### Literature Quality Assessment

An assessment of literature quality was performed. All of the final 22 publications were independently reviewed and scored by two reviewers. The assessment tool for blockchain-related studies proposed by Petersen et al [12] was used to critically appraise and summarize evidence in the searched papers.

In accordance with Hölbl et al [13], the quality of the papers was assessed using the criteria defined in Table 1.

This tool was used to assess the trustworthiness, relevance, and results of the published papers. These led to the decision of which papers were believable and useful and could be used for the research. A three-tier scale was used to rank the quality of all four questions. A value of 0 (“barely” or “no”) was assigned when the criterion was addressed very poorly or not at all, a value of 1 (“partially”) was assigned when a criterion was partially addressed, and a value of 2 (“satisfactorily” or “yes”) was assigned when the reviewer felt that the publication had successfully satisfied the criterion.

Two reviewers assessed each query from question 1 (Q1) to question 4 (Q4), which resulted in a minimum of 0 points to a maximum of 4 points per query. The minimum score for the sum of responses to question 2 (Q2), question 3 (Q3), and Q4 was 0 points, and the maximum score was 12 points. The score of the response to Q1 was converted to 40% of the total value, and the total summed score of responses to Q2, Q3, and Q4 was converted to 60% of the total value, with Q2, Q3, and Q4 each contributing 20% of the total points.

So, the overall score was the sum of responses to Q1 to Q4, which is presented as a percentage to enhance readability and comprehension. An explanation of scoring of responses to queries Q1 to Q4 is given in Table 1.

To find the percentage score of the response to Q1, the following equation applies:



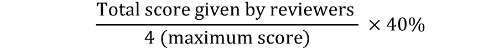



To find the percentage score for the sum of responses to Q2 to Q4, the following equation applies:



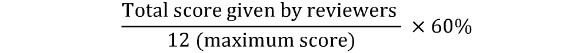



The overall score is represented by the following equation: Overall score = Percentage score of response to Q1 (%) + Percentage score of sum of responses to Q2 to Q4 (%) [13].

From the calculation, the publications that have an overall score of 90% and above are high-quality papers. An overall score between 80% and 89% indicates a moderate-quality paper, and low-quality papers are represented by an overall score of 79% or less.

**Table 1:** Parameters for measuring quality of the publications [13].

Question (Q)	Quality assessment query	Responses (scores)
Q1	Is the publication relevant to blockchain?	“barely” (0), “partially” (1), or “satisfactorily” (2)
Q2	Does the publication include and define research objectives adequately?	“no” (0), “partially” (1), or “yes” (2)
Q3	Are limitations and challenges well defined?	“no” (0), “partially” (1), or “yes” (2)
Q4	Is the proposed contribution well described?	“no” (0), “partially” (1), or “yes” (2)

### Data Availability

All data have been reported in this manuscript.

## Results

### Study Selection

A total of 271 papers were initially retrieved as a result of implementation of the search protocol that was designed for searching the selected scientific databases. Of the 271 papers, 34 (12.5%) were from PubMed, 52 (19.2%) were from SpringerLink, 40 (14.8%) were from IEEE Xplore, 56 (20.7%) were from Embase, 45 (16.6%) were from Scopus, and 44 (16.2%) were from *EBSCOhost*. Then, the first screening was done based on the titles of the retrieved papers. All of the paper titles were examined independently by one reviewer based on the inclusion and exclusion criteria, which led to the selection of 175 papers. In the first screening, a total of 52 papers were excluded because they were not related to the research topic (eg, some excluded papers discussed the business perspective of Bitcoin rather than the use of blockchain technology in the health care sector). Meanwhile, 25 papers related to other scientific areas, such as mathematics and chemistry, were excluded from the first screening, as the term “blockchain” had other meanings apart from the technology used in computer science and IT. Through a manual search and using references from the included papers, an additional 10 papers were collected.

After the selection of 185 papers from the first screening, 87 duplicate papers were removed using Endnote X8. This resulted in 98 papers, which underwent further screening based on abstracts where, in some cases, the introduction and conclusion of the full text were analyzed. The abstracts of all the selected papers were read by two reviewers. Some of the papers were removed because the abstracts indicated no relevance to the research topic. The unclear or grey-area abstracts or papers were moved to the next screening step for more in-depth analysis.

**Figure 1:**
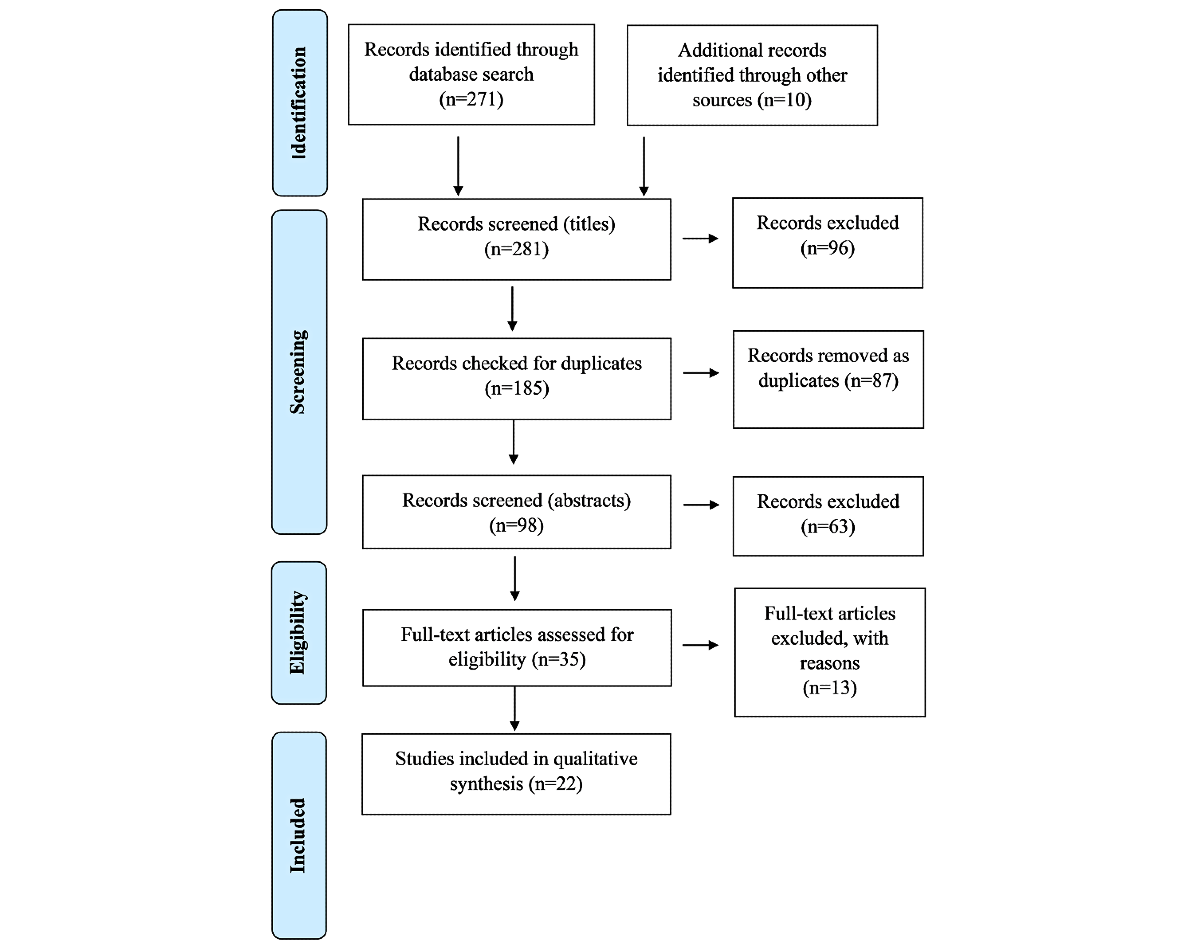
PRISMA (Preferred Reporting Items for Systematic Reviews and Meta-Analyses) flowchart of the search strategy.

A total of 35 papers were identified for full-paper analysis, which was the last stage of paper selection for this systematic review. Each paper was read in full, independently, and assessed for eligibility using the inclusion and exclusion criteria. This resulted in the selection of 22 primary papers. Of the 35 papers identified for full-text analysis, 3 (9%) were dropped because they focused on the economic perspective of Bitcoin and not the health care setting. Of the 35 papers, 5 (14%) were removed as they only described blockchain and how it works, without discussing any actual blockchain implementation in a real health care environment. Furthermore, 3 out of 35 (9%) papers identified as review papers and 2 (6%) papers identified as proposal papers were removed. Figure 1 shows the results of the search strategy. The list of 22 selected primary papers and the extracted data items are included in Table 2 [14-35]. The PRISMA (Preferred Reporting Items for Systematic Reviews and Meta-Analyses) checklist for this research study on the use of blockchain technology in the health care sector is included in Multimedia Appendix 2.

**Table 2:** Information extracted and collected from the selected papers.

Study first author, year	Location	Use cases and fields		Usability and reasons for using blockchain		Technology
		Application area	Description	Simplified classification	Description	
Maslove, 2018 [14]	Kingston, Canada	Biomedical research and education (ie, clinical trials)	Development of a system that uses a web-based interface to allow users to run trial-related smart contracts on an Ethereum network	Data integrity	Enabling of clinical trials data management; functions allow patients to grant researchers access to their data and allow researchers to submit queries for data that are stored off-chain	Ethereum
Cunningham, 2017 [18]	The Netherlands	Electronic medical record (EMR)	A system that uses smart contract–based Ethereum blockchain technology to operate in a verifiably secure, trustless, and openly auditable environment	Access control	Improvement of the uptake and acceptance of medical informatics platforms where patients directly control medical data in an open and secure manner	Ethereum
Nugent, 2016 [29]	London, the United Kingdom	Biomedical research and education (ie, clinical trials)	A system that uses smart contracts, which enhance the trust in the data and clinical trials; this reduces patient risk and financial strain in health care by allowing better-informed decisions to be made by medical professionals	Data integrity and logging	Improvement of data transparency in clinical trials and immutable records of trial history, which act as trusted administrators; tamper-resistant characteristics of blockchain prevent all forms of manipulation; mainly used for complex clinical trial management	Ethereum
Benchoufi, 2017 [15]	Paris, France	Biomedical research and education (ie, clinical trials)	A system with time-stamping of each patient’s consent using blockchain technology in a securely unfalsifiable and transparent way	Nonrepudiation, logging, and data versioning	All consent-related data on the blockchain enhance security, reliability, and transparency and could be a consistent step toward reproducibility	N/A^a^
Ichikawa, 2017 [16]	Tokyo, Japan	Remote patient monitoring (ie, mobile health [mHealth])	Development of a smartphone app with blockchain technology to provide an mHealth system for cognitive behavioral therapy for insomnia	Data integrity	Establishment of accessibility and transparency of data without the third party by incorporating blockchain technology into mHealth; blockchain also serves as a tamperproof system for mHealth	Hyperledger Fabric
Cichosz, 2018 [25]	Aalborg, Denmark	EMR (ie, health care data)	Development of a platform using the New Economy Movement (NEM) multi-signature blockchain contracts to access data management, sharing, and encryption	Access control and data integrity	Improvement in privacy and diabetes data management, where patients have access to control and share their own data	NEM
Omar, 2019 [26]	The United States	EMR	Development of a patient-centric health care data management system using blockchain technology as storage, which enhances privacy	Data integrity	Patients will have overall control over their data; MediBchain increases patients’ interest in EMRs or electronic health records and enhances accountability, integrity, pseudonymity, security, and privacy	N/A
Liang, 2017 [30]	Norfolk, England	Remote patient monitoring	Development of an mHealth care system for personal health data collection, sharing, and collaboration between individuals, health care providers, and insurance companies, and its implementation in a distributed and trustless way	Data integrity, access control, and logging	Improvement of personal health data collection, sharing, validation, protection, and integrity and health care collaboration; this system ensures the scalability and efficiency of the data process by handling a large data set at low latency	Hyperledger Fabric
Kleinaki, 2018 [28]	The Netherlands	Biomedical research and education (ie, database queries)	Presentation and testing of the use of smart digital contracts by a blockchain-based notarization service to seal a biomedical database query using a real blockchain infrastructure	Data integrity and data versioning	Improvement of retrieved data integrity, nonrepudiation, and biomedical evidence data versioning	Ethereum
Bocek, 2017 [32]	Zurich, Switzerland	Pharmaceutical supply chain (ie, ambient temperature)	Implementation of sensor devices using blockchain technology to enhance data immutability and public accessibility of temperature records	Logging	This system can be evaluated automatically, and the stored data are tamperproof with Ethereum, which can be used at a low cost	Ethereum
Mendes, 2018 [22]	Évora, Portugal	EMR	Development of a system with raw blockchain with Hyperledger Fabric by DLA	Data integrity	While consuming low computational power, it enhances tamperproof, fair, and democratic maintenance of the ledger	Hyperledger Fabric
Li, 2018 [20]	Beijing, China	EMR (ie, health record)	Development of a novel blockchain-based data preservation system based on the real-world blockchain-based platform, and its implementation for medical data	Data integrity	Preservation of important data in perpetuity and verification of data originality; illegal operation of the data is detected, and the user is notified on time	Ethereum
Azaria, 2016 [17]	The United States	EMR (ie, health record)	Development of a decentralized record management system using blockchain technology to handle EMRs	Logging and access control	The system becomes more convenient and adaptable in its management of authentication, confidentiality, accountability, and data sharing	Ethereum
Zhou, 2018 [33]	Beijing, China	Health insurance claims	Development of a blockchain-based medical insurance storage system, MIStore; this helps insurance companies obtain patients’ medical spending records, which are always confidential	Data integrity and logging	The system provides decentralization and tamper resistance; this gives users high credibility and record-nodes, which help users verify publicly verifiable data	Ethereum
Angeletti, 2017 [27]	Rome, Italy	Biomedical research and education (ie, clinical trials)	Presentation of a digital health application enabling clinical trials recruitment using Internet of Things data; using Ethereum, a proof of concept was implemented, and the application’s performance was studied in a real-world evaluation	Data integrity and access control	The clinical research institute can be guaranteed that it is acquiring useful and original data; until an agreement is reached, the individual can keep personal data private	Ethereum
Saravanan, 2017 [31]	Chennai, India	Remote patient monitoring	Implementation of a new health care paradigm (SMEAD^b^) to aid diabetic patients via development of an end-to-end secured system; implementation of a blockchain-based disruptive technology to facilitate cryptographic security and formalized data access through smart contracts	Access control	The system aids in data storage for millions of patients, and analysis was performed in real time, which promotes an evidence-based medicine system with privacy and security concerns	Ethereum
Zhang, 2018 [24]	The United States	EMR (ie, health record)	Development of a system to support collaborative clinical decision-making via a remote tumor board case study	Access control and data integrity	Improvement of security, trust, and scalable data sharing, which is important for collaborative clinical decision-making; also results in greater data readability	Ethereum
Fan, 2018 [19]	China	EMR (ie, health record)	Development of a blockchain-based information management system, MedBlock, to handle patients’ information; this allows for efficient EMR access and retrieval, exhibiting high information security	Access control	Patients can easily access the EMRs of different hospitals; data sharing via blockchain helps the hospital get a full history of patients’ medical history before consultations are carried out	N/A
Liu, 2018 [21]	China	EMR	Implementation of blockchain-based privacy-preserving data sharing for EMRs	Access control	The EMRs cannot be modified arbitrarily, which leads to reduced medical data leakage; security analysis shows that this system is a secure and effective way to realize data sharing for EMRs	N/A
Nagasubramanian, 2018 [23]	London, the United Kingdom	EMR	Ensuring secrecy of digital signatures and authentication by using keyless signature infrastructure in the system	Access control	The system ensures data transparency, privacy, confidentiality, and verification of data	N/A
Kotsiuba, 2018 [34]	Ukraine	Health care data analytics	Implementation of a decentralized system with blockchain technology that protects the confidentiality of medical data; patients receive a personal data monitoring tool, allowing them to participate in accelerating medical analytics	Data integrity	Enhancement of medical data safety, extension of the base of clinical data collection, and creation of an effective shared health infrastructure	N/A
Talukder, 2018 [35]	The United States	Others	Implementation of an Ethereum-based Proof of Disease consensus protocol to enhance the accuracy of transactions and eliminate medical errors	Access control and data integrity	Aids in achieving all the complex needs of P6 (participatory, personalized, proactive, preventive, predictive, and precision) medicine and decreases disease burden	Ethereum

^a^N/A: not applicable: the technology was not reported in this paper.

^b^SMEAD: Secured Mobile-Enabled Assisting Device for Diabetics.

### Publication Year, Publication Type, and Geographical Distribution

All the selected papers were published since 2016. This indicates that blockchain technology in health care settings is very new. It is noted that from the 22 selected papers, the majority (n=12, 55%) were published in 2018, 7 (32%) papers were published in 2017, 2 (9%) papers were published in 2016, and 1 (5%) paper was published in 2019.

The locations (ie, countries) of the institutions of the authors of the selected primary papers were used to distinguish the geographical distribution of the research community members who were involved in the research. If a paper had authors from different countries, the country of the corresponding author was used. It is noted that the authors, universities, and companies in the United States and China were leading, having 4 (18%) papers each. This was followed by the United Kingdom with 3 (14%) papers and the Netherlands with 2 (9%) papers. The rest of the countries, namely Ukraine, India, Italy, Portugal, Switzerland, Denmark, Japan, France, and Canada, contributed 1 (5%) paper each. This geographical distribution of the 22 selected papers indicates that blockchain technology in the health care sector has gathered research interest around the world.

The channels where the papers were published determined the publication type. The two publication types included in this systematic review were conferences and journals. The majority (n=15, 68%) of the 22 selected primary papers were published in journals, while 7 (32%) were published as conference proceedings.

The 22 selected primary papers were studied, and the data or keywords related to this systematic review’s question were extracted. A classification scheme was constructed based on the iterative identification of data, charting keywords extracted from the selected papers. The papers were then sorted into identified categories.

Each of the selected primary papers addressed one or more different aspects of the use cases of blockchain technology in the health care sector. Therefore, the identified use cases were used to further classify the selected papers. Out of 22 selected papers, 10 (45%) addressed the application of blockchain in the management of EMRs, 5 (23%) addressed the use of blockchain technology in biomedical research and education, and 3 (14%) demonstrated the use of blockchain technology in remote patient monitoring. The remaining papers addressed the use of blockchain technology in drug or pharmaceutical supply chains (n=1, 5%), health insurance claims (n=1, 5%), health data analytics (n=1, 5%), and other applications (n=1, 5%).

In the selected primary papers, blockchain was implemented in the real health care environment to address several information security components. The use of blockchain technology or the main reason it was implemented in health care was classified. From this data, it was noted that each of the selected primary papers addressed one or more reasons, out of a total of 34 reasons or benefits, for using blockchain technology in health care. Most papers addressed the application of blockchain in health care for data integrity (14/34, 41%). The next largest purpose of blockchain application was access control, which contributed 11 out of 34 (32%) reasons in the papers. Meanwhile, data logging was addressed 6 (18%) times, data versioning was addressed 2 (6%) times, and nonrepudiation was addressed 1 (3%) time.

The setting of the studies, specifically the type of hospital used for the implementation of blockchain technology, was analyzed among the 22 selected papers. Only 3 out of 22 (14%) papers gave the name of hospital where the study was carried out. Maslove et al [14] implemented a blockchain-based smart contract at Kingston General Hospital, Canada, to study how blockchain technology could be used in clinical trial data management, which could enhance data integrity. Another study on clinical trials data management was conducted by Benchoufi et al [15] at Hospital Hôtel Dieu, Paris, France, which looked at whether the implementation of blockchain could enhance the transparency and traceability of clinical trial consent, thereby benefitting both patients and researchers. Ichikawa et al [16] conducted their study at the Institute of Neuropsychiatry, Seiwa Hospital, Tokyo, Japan, using blockchain technology to implement a tamper-resistant mobile health (mHealth) system, which could enhance both data transparency and accessibility without the involvement of a third party.

Information regarding the blockchain platform that was used was gathered from the 22 selected papers. Ethereum was the most commonly used blockchain platform (n=12, 55%), followed by Hyperledger Fabric (n=3, 14%), while the least used platform was the New Economy Movement (NEM) blockchain platform (n=1, 5%). The rest of the studies did not state which blockchain platform was used.

### Literature Quality Analysis

The final and crucial part of this systematic review involved reviewers scoring the 22 papers to evaluate their quality and the relevance of the blockchain usage. The scoring results are shown in Table 3 [14-35]. These show a greater quality of average overall scores among 15 journal papers (mean score 81.0%, SD 5.8%) compared to 7 conference papers (mean score 77.1%, SD 5.9%). Out of the 22 papers, 3 (14%) high-quality papers, 9 (41%) moderate-quality papers, and 10 (45%) low-quality papers were identified. It is noted that no published conference papers from January to September 2019 were found or included in this study.

The 2 (9%) papers published in 2016 each had a relatively high average overall score. In 2017, the average overall score of the papers was 80%, which was slightly lower than that of 2016. This dropped to 78% in 2018 and rose again to 80% in 2019.

**Table 3:** Summary of scores for measuring quality of the publications.

Study first author, year	Type of publication	Points per question (Q)^a^					Sum of scores for Q2-Q4		Overall score for Q1-Q4 (%), mean (SD)		Quality of paper^b^
		Q1	Q2	Q3	Q4						
Maslove, 2018 [14]	Journal	4	3	3	4	10		90.0 (0.5)		High	
Benchoufi, 2017 [15]	Journal	4	3	2	3	8		80.0 (0.7)		Moderate	
Ichikawa, 2017 [16]	Conference proceeding	4	4	2	3	9		85.0 (0.8)		Moderate	
Azaria, 2016 [17]	Journal	4	3	2	3	8		80.0 (0.7)		Moderate	
Cunningham, 2017 [18]	Journal	4	3	2	4	9		85.0 (0.8)		Moderate	
Fan, 2018 [19]	Journal	4	3	1	3	7		75.0 (1.1)		Low	
Li, 2018 [20]	Conference proceeding	4	3	2	4	9		85.0 (0.8)		Moderate	
Liu, 2018 [21]	Journal	4	3	2	2	7		75.0 (0.8)		Low	
Mendes, 2018 [22]	Journal	4	3	1	3	7		75.0 (1.1)		Low	
Nagasubramanian, 2018 [23]	Journal	4	3	2	3	8		80.0 (0.7)		Moderate	
Zhang, 2018 [24]	Journal	4	3	1	3	7		75.0 (1.1)		Low	
Cichosz, 2018 [25]	Journal	4	4	2	3	9		85.0 (0.8)		Moderate	
Omar, 2019 [26]	Conference proceeding	4	3	1	3	7		75.0 (1.1)		Low	
Angeletti, 2017 [27]	Journal	4	4	2	4	10		90.0 (0.9)		High	
Kleinaki, 2018 [28]	Journal	4	3	1	3	7		75.0 (1.1)		Low	
Nugent, 2016 [29]	Journal	4	4	2	4	10		90.0 (0.9)		High	
Liang, 2017 [30]	Conference proceeding	4	3	1	3	7		75.0 (1.1)		Low	
Saravanan, 2017 [31]	Journal	4	3	1	3	7		75.0 (1.1)		Low	
Bocek, 2017 [32]	Journal	4	4	1	4	9		85.0 (1.3)		Moderate	
Zhou, 2018 [33]	Conference proceeding	4	4	1	3	8		80.0 (1.2)		Moderate	
Kotsiuba, 2018 [34]	Conference proceeding	4	3	1	2	6		70.0 (1.1)		Low	
Talukder, 2018 [35]	Conference proceeding	4	3	0	3	6		70.0 (1.5)		Low	

^a^Two reviewers assessed each query from Q1 to Q4, based on a 5-point ordinal scale ranging from 0 to 4, where 0 indicates the lowest level (criterion was addressed very poorly or not at all) and 4 indicates the highest level (criterion was exceptional).

^b^An overall score of ≥90% indicates a high-quality paper; an overall score of 80%-89% indicates a moderate-quality paper; an overall score ≤79% indicates a low-quality paper.

## Discussion

### Identified Themes

**Figure 2:**
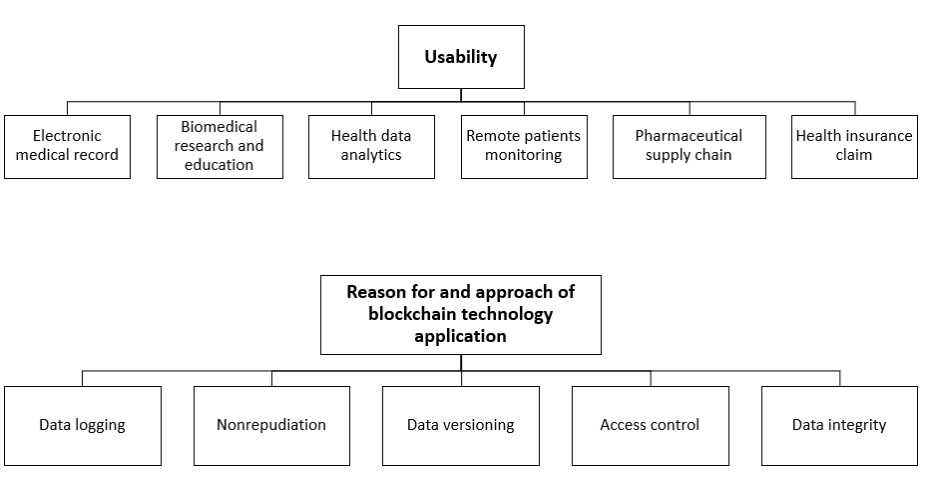
The uses of blockchain technology in the health care sector.

The identified themes are summarized in Figure 2.

### The Use of Blockchain Technology in Real Health Care Environments

The results from this systematic review show that the majority of the research regarding blockchain technology in health care environments was focused on the management of EMRs, followed by biomedical research and education, remote patient monitoring, pharmaceutical supply chains, health insurance claims, health data analytics, and other potential areas.

### Electronic Medical Records

Out of 22 selected papers, 10 (45%) concentrated on the management of EMRs. EMRs, similar to EHRs or personal health records, involve electronic modeling, storage, and management of patients’ personal, medical, or health-related data. Traditionally, different systems have been used to store patients’ records separately across different service providers, where the service providers have control over the records, which may limit data sharing with other health care stakeholders.

The application of blockchain in the management of EHRs will make data sharing among health care stakeholders easier, more transparent, and more trustworthy, and patients will have control over their own data. This is because the characteristics of blockchain technology, such as decentralization, immutability, data provenance, reliability, robustness, smart contracts, security, and privacy, make it suitable for the management and storage of patient EHRs [18].

Azaria et al [17] presented MedRec, which is a project from the MIT (Massachusetts Institute of Technology) Media Lab and Beth Israel Deaconess Medical Center that uses a blockchain-based platform to give patients access to their own data through some access permissions built into the blockchain. The patient may decide to grant access to their EHRs to any third party, which may reduce their paperwork, given that patients normally have to carry a bundle of papers with them when they seek out different health care providers for consultation. With blockchain technology, regardless of the time and institution, health care providers can easily gain access to all of a patient’s medical data. Patients become more committed to their own health care because they are directly involved in the management of their health records through blockchain technology.

The second application that would integrate EMRs is blockchain-based privacy-preserving data sharing (BPDS), which was developed by Liu et al [21]. This uses the Ethereum blockchain platform, which reduces the risk of medical data leakage and secures data sharing in health care.

Fan et al [19] developed MedBlock, a blockchain-based information management system that is implemented in health care to enhance efficiency and secure electronic medical data sharing using blockchain. Another blockchain-based EMR is FHIRChain [24], which encapsulates the Health Level Seven Fast Healthcare Interoperability Resources (FHIR) standard for shared clinical data. Zhang et al [24] used blockchain technology via the FHIRChain-based decentralized app to share clinical data that focused on health care record management and digital health identities to verify participants for remote cancer care in a case study of collaborative decision-making [24]. Cichosz et al [25] proposed NEM multi-signature blockchain contracts to be used for the management and sharing of medical data of diabetes patients, which aimed to achieve access control and data privacy.

Li et al [20] presented a medical data preservation system based on the real-world blockchain platform Ethereum, which provides a trustworthy storage solution to ensure the primitiveness and verifiability of stored data. Mendes et al [22] presented a Smart Ambient Assisted Living environment, which uses blockchain technology to enhance data privacy and cognitive security in the health care sector. As noted above, out of the 22 included papers, 3 (14%) were high-quality papers, 9 (41%) were moderate-quality papers, and 10 (45%) were low-quality papers. Therefore, the conclusions made are convincing based on the high or moderate quality of a high percentage of papers.

### Biomedical Research and Education: Clinical Research

A total of 23% (5/22) of the selected papers in this study indicated that blockchain could be applied in biomedical research and education fields. Blockchain technology has been used extensively in biomedical research and education to preserve data privacy, integrity, sharing, record sharing, and record keeping, especially in clinical trials [23]. Nugent et al [29] presented blockchain smart contracts that prevent falsification of data and underreporting of unwanted results of clinical research, which enhances trust in the data and clinical trials.

Angeletti et al [27] proposed a proof-of-concept implementation of consent traceability in clinical trials using Ethereum to secure and ensure unfalsifiable data. Every piece of data or consent included in the blockchain system is time-stamped and publicly transparent. This is achieved through cryptographic validation. All plans, consent, protocols, and possible outcomes can be stored on blockchain even before the inception of clinical trials, which prevents any corruption and undesirable study results.

Kleinaki et al [28] presented a blockchain-based notarization service that uses smart contracts to seal biomedical database queries and the respective results, which ensures data transparency. Maslove et al [14] proposed BlockTrial, a web-based interface system that allow users to run trial-related smart contracts on the Ethereum network in clinical data management, thereby enhancing the reliability and transparency of complex data in clinical trials. The tamperproof characteristics of blockchain prevents the manipulation of data in clinical trials.

### Remote Patient Monitoring

Remote patient monitoring was another blockchain use case in the health care sector. Generally, remote patient monitoring includes the gathering of biomedical data from the body and mobile devices to enable the monitoring of patient status remotely outside of traditional health care environments, such as hospitals.

Liang et al [30] presented a Hyperledger-based implementation of blockchain in mHealth that enables data collection and sharing between health care stakeholders, ensuring both data transparency and accessibility. Saravanan et al [31] proposed an end-to-end secured system, a new health care paradigm (ie, Secured Mobile-Enabled Assisting Device for Diabetics), through smart contracts to facilitate cryptographic security and formalized data access in which to monitor diabetes patients. The author stated that blockchain was engaged in a mobile-enabled assisting device that was developed to monitor diabetes patients. Ichikawa et al [16] presented a tamper-resistant mHealth system using blockchain technology where a mobile device is used to gather EMRs, which are then sent to the blockchain-based Hyperledger Fabric network to ensure secure management of the data.

Cichosz et al [25] proposed NEM multi-signature blockchain contracts for assisting diabetes patients in monitoring and transmitting their vital parameters or data by sensor device to a blockchain-based platform where the data are collected, stored, and analyzed. In emergency cases, such as abnormal blood glucose levels or missing dosages, an alert via a social network, such as Facebook or WhatsApp, will be sent to the care provider. The data can be communicated continuously by using mobile devices as a gateway with blockchain technology, which could save patients from any untoward consequences.

### Drug or Pharmaceutical Supply Chains

Drug or pharmaceutical supply chains are one of the use cases of blockchain technology in the health care sector, particularly health-related supply chain management. Drug or pharmaceutical supply chains involve the introduction of new drugs into the market, ensuring the safety and validity of medical products sold to end customers [32]. Blockchain has been applied in this field to allocate a safe and secure platform and to address the most common problems faced in the pharmaceutical industry, such as delivery of substandard or counterfeit medication, which may have a negative impact on patients.

In this systematic review, only 1 paper out of 22 (5%) presented the implementation of a blockchain-based application for pharmaceutical supply chain management. Bocek et al [32] presented a real-world demonstration and evaluation of blockchain technology in the pharmaceutical supply chain, where ambient temperature sensors with blockchain technology were used to record temperatures at which drugs were stored and transported; such temperature measurements were immutably kept in a public blockchain for transparent inspection, which could also decrease the operational cost in a pharmaceutical supply chain.

### Health Insurance Claims

Health insurance is necessary for everyone to get affordable medical treatment. Blockchain’s characteristics, such as immutability, decentralization, transparency, and auditability of records, can benefit the process of health insurance claims in the health care sector. Nevertheless, only 1 paper from the 22 (5%) selected primary papers focused on this application. Zhou et al [33] developed a blockchain-based medical insurance storage system that is displayed using the Ethereum blockchain platform. The medical insurance data of a patient can be encrypted and immutably stored on blockchain, which enhances credibility and eliminates the involvement of third parties in the management of patients’ health insurance [33].

### Health Data Analytics

Only 1 paper out of 22 (5%) presented the use of blockchain technology in health data analytics. Blockchain in collaboration with other emerging technologies, such as deep- and transfer-learning techniques, was used to identify predictive analytics of health care data. Kotsiuba et al [34] stated that blockchain provides a unique opportunity to overcome the problems related to the analysis and security of medical data. Using blockchain technology, a decentralized health data ecosystem was presented that protected medical data confidentiality, produced an effective shared health infrastructure, and increased the basis of clinical data collection.

### Other Functionalities

Of the 22 selected primary papers, 1 (5%) study by Talukder et al [35] included the relevant research perspective but could not be classified under any of the identified uses of blockchain. This study presented a blockchain consensus protocol that provides accurate medical decisions and reduces the disease burden by using Ethereum, based on the proof-of-disease consensus protocol. All functionalities of health data interoperability, including EMRs, patient health records, and health information exchange databases, can be achieved by this system.

### Reasons for the Application of Blockchain Technology in the Current Health Care System

#### Overview

The main reasons (N=34) for the application or implementation of blockchain technology in the health care sector in this study’s selected papers were identified and categorized into the following groups: data integrity (n=14, 41%), access control (n=11, 32%), data logging (n=6, 18%), data versioning (n=2, 6%), and nonrepudiation (n=1, 3%).

#### Data Integrity

Data integrity is defined as the accuracy and consistency of the data or information stored in a system, which acts as an important component of information security. Data integrity was achieved by using blockchain technology in the health care sector. Li et al [20] implemented the blockchain-based platform Ethereum to maintain the originality and variability of stored data in the system while preserving user privacy. The lifelong maintenance of data in blockchain was achieved with the proof-of-primitiveness data concept, and the system can validate the data where it is identical to the original data. The data can be restored and verified through blockchain if it has been damaged.

Kotsiuba et al [34] also presented a decentralized health data system using blockchain that secures the collection and confidentially of medical or clinical data. In the study by Zhang et al [24], data integrity was enhanced by using an FHIRChain-based decentralized app, which used blockchain technology and digital health identities in remote cancer care to validate the participants in a case study of clinical data sharing. With the application of public key cryptography, this decentralized app improves the trust of participants and enables the users to share specific and structured pieces of information, rather than an entire document. Thereby, it increases the readability of data and flexibility of sharing options.

Cichosz et al [25] implemented a blockchain-based platform to enhance the management and sharing of diabetes data in an easy and secure way, which can be achieved by a decentralization of blockchain. According to a study by Omar et al [26], the integrity, security, privacy, and accountability of data in health care are achieved through a privacy-preserving platform using blockchain technology. To ensure encryption of patient data and pseudonymity, a cryptographic function was used. The decentralization of data enabled by the peer-to-peer network in blockchain technology helps to reduce cyberattacks and preserve the health care data set.

The proof-of-concept implementation of patient-facing and researcher-facing systems using blockchain technology to enhance data integrity was demonstrated by Maslove et al [14] and Angeletti et al [27]. Maslove et al [14] demonstrated that the proof-of-concept implementation using blockchain technology in clinical trials secures original personal data, and this data would not be shared publicly before an agreement is reached. In regard to the use of blockchain technology in clinical trials, the clinical research institute can also guarantee that the data obtained are authentic and useful.

Angeletti et al [27] stated that the integrity of the data collected in clinical trials was enhanced by the application of blockchain technology, specifically blockchain-based smart contracts, which act as the foundation to promote trust throughout clinical research. The proof-of-concept implementation in clinical research enhances the interaction of researchers and patients.

Blockchain in digital health technologies has also been particularly used in mHealth, which includes remote patient monitoring to ensure the safe and precise preservation of medical information to improve data integrity. Ichikawa et al [16] concluded from their study that the usage of blockchain technology in mHealth improves data transparency and accessibility without the involvement of third parties, due to the tamperproof and decentralized characteristics of blockchain technology.

#### Access Control

According to Azaria et al [17], access control is defined as an individual having full authority in deciding who can access their medical data, as well as when and how much of their own medical data can be accessed using blockchain technology. Access control may lead to patients’ direct involvement in controlling their own medical data usage. The distributed ledger, which is one of the characteristics of blockchain technology, ensures efficient access and retrieval of EMRs [18].

Fan et al [19] used a proof of concept with an application programming interface using blockchain technology, which allows a permission system where each patient is able to view, control, and specify who can access their records.

A study by Cunningham and Ainsworth [18] found that the EMRs that included a patient’s full medical history from many different hospitals could be easily accessed by the patient using a blockchain-based information management system, which enhances the outcome of treatment by avoiding the segregation of medical data from different hospitals. An access protocol was implemented that prevented unauthorized users from obtaining any sensitive data or information.

For access control and the preservation of data, Fan et al [19] used the blockchain-based platform concept with NEM multi-signature blockchain contracts, which ensured privacy control of health data. With this concept, patients are in control of their own data and have the power to decide who can access their personal data. For instance, an older adult patient could share access of their medical data with their adult child.

According to Cichosz et al [25], patients were able to access their own medical data through smart blockchain contracts, which may lead to secure data sharing. Through BPDS, which consists of data access permission implemented by Liu et al [21], patients have full control over their medical records or data, without jeopardizing their privacy. Furthermore, a user can use patient data with permission from the patient. The owner of the data in blockchain is capable of revoking his or her access permission, in case of a violation of access rules.

According to Liu et al [21], health records that are centrally stored are more vulnerable to cyberattacks. Therefore, Nagasubramanian et al [23] presented a keyless signature infrastructure (KSI) blockchain technology for securing EHRs that ensures authentication and integrity of health records. In a KSI blockchain system, the signed data are stored and can be operated without a network connection, and no third parties are required to preserve data in this system.

Data access by health care professionals can be achieved through smart contracts of blockchain technology with cryptographic security. Using blockchain technology, Saravanan et al [31] implemented a mobile-based secure health care system that can predict a patient’s diabetes status in real time. In case of emergency, the doctor can access a patient’s health record and prescribe them with a suitable medication dosage using this technology system. This blockchain system is used to store data related to health care and securely connect with third parties.

#### Data Logging

Data logging is defined as an operation of gathering and storing information over a period of time. It allows tracking of all types of interactions, such as storage, access, or modification of data, files, or applications in a system. Data logging can be achieved by the application of blockchain technology in the health care sector.

In clinical trials, Nugent et al [29] demonstrated blockchain technology using an Ethereum smart contract to enhance the trustworthiness, reliability, and transparency of data management. The cryptographic and tamperproof characteristics of blockchain prevent all forms of manipulation and enhance the data logging of complex clinical trial data management, so more informed decisions can be made by medical professionals. An mHealth care system using blockchain technology was implemented by Liang et al [30] that ensured gathering, sharing, and collaboration of data between the health care providers and individuals in a secure way.

Bocek et al [32] stated in their study that the application of blockchain technology in pharmaceutical supply chain management ensures data logging. They demonstrated the use of an Internet of Things sensor device (modum.io AG) that uses blockchain technology to ensure the verification of compliance with quality control temperature requirements. This device was used to monitor and store the temperature of products, enhance data immutability, and facilitate public accessibility of temperature records of pharmaceutical products, especially during transportation. Data provenance was ensured using blockchain technology that can prove the origin of products in a supply chain.

Zhou et al [33] stated that blockchain technology acts as a tamperproof and decentralized technology to record data, which enhances users’ trust in a health insurance system, especially with the implementation of a blockchain-based medical insurance storage system. For instance, the data about each patient’s spending was stored and secured in the blockchain by the hospital, which helped the insurance company obtain information about the total amount of spending by the patient; however, third parties, including the insurance company, cannot modify or delete the data and do not have the authority to access a patient’s personal medical data.

#### Data Versioning

Data versioning is defined as saving new copies of the data when any modification is made to the existing data. This helps to keep track of the data and ensure easy retrieval of any specific version of the respective stored data in a system. Kleinaki et al [28] implemented a blockchain-based notarization service that uses smart digital contracts to secure data in the biomedical research sector. A study by Mendes et al [22] showed that after the retrieval process, retrieved data cannot be modified, which ensures the integrity and nonrepudiation of the data. Using blockchain technology, data versioning was achieved where medical evidence of different versions of data retrieved from a biomedical database were securely stored and saved, along with content that is continually updated. In this study, this was mostly used for decision support in the health care sector.

#### Nonrepudiation

Nonrepudiation guarantees the validity of data in a particular health care system, which cannot not be denied by anyone and ensures the originality and integrity of data. A study by Angeletti et al [27] used blockchain technology to collect, store, and track clinical trial consent in a secure, unfalsifiable, and publicly verifiable way; this consent was originally time-stamped with the application of proof of concept, leading to the nonrepudiation of data. The authentication system ensures that the clinical trial consent is accessible and transparent for patients, while traceable for stakeholders. A single document in open format was used and accounted for the whole time-stamped consent collection process. This document cannot be corrupted and is considered a robust proof of data.

### Study Limitations

One limitation of this systematic review study was that there were no published studies on the safety of blockchain technology in health care, so the safety aspect of blockchain technology cannot be reviewed. In addition, there were few papers published on the negative aspects of implementation of blockchain technology in health care. Most studies only published the positive aspects, which may have led to bias.

### Future Directions

Blockchain technology is still a new technology that has not been widely implemented in the health care sector. This study can be a guide for future research, implementation, and evaluation of blockchain technology in this sector. More research should be carried out regarding the implementation of blockchain technology in real health care environments for better understanding, characterization, and evaluation. Researchers should also focus on carrying out research on the safety of implementing blockchain technology in health care.

### Conclusions

This systematic review has presented an overview of the use and characteristics of blockchain technology in the health care sector. The findings show that blockchain technology research and application in the health care sector is still in its infancy but growing rapidly. Blockchain technology has started to develop from cryptocurrencies, such as Bitcoin, into various general-purpose technologies in many industries, including health care. According to the selected papers in this study, EMRs, biomedical research and education, remote patient monitoring, drug or pharmaceutical supply chains, health insurance claims, and health data analytics are the most common uses of blockchain technology in health care. The main reasons for the application of blockchain technology are to enhance data integrity, access control, logging, data versioning, and nonrepudiation of patient health records or other health information in health care settings.

## Appendix

Multimedia Appendix 1 Databases and search terms used.

Multimedia Appendix 2 PRISMA (Preferred Reporting Items for Systematic Reviews and Meta-Analyses) checklist for the systematic review of the use of blockchain technology in the health care sector.

## Abbreviations

BPDS: blockchain-based privacy-preserving data sharing
EHR: electronic health record
EMR: electronic medical record
FHIR: Fast Healthcare Interoperability Resources
HI: health informatics
HIS: hospital information system
IT: information technology
KSI: keyless signature infrastructure
MeSH: Medical Subject Headings
mHealth: mobile health
MIT: Massachusetts Institute of Technology
NEM: New Economy Movement
PRISMA: Preferred Reporting Items for Systematic Reviews and Meta-Analyses
Q1: question 1
Q2: question 2
Q3: question 3
Q4: question 4
