# Supplementary material for: The Use of Blockchain Technology in the Health Care Sector: Systematic Review
Source: JMIR Med Inform. 2022 Jan 20;10(1):e17278. doi: 10.2196/17278 (PMC8814929; doi:10.2196/17278)
Supplement: Multimedia Appendix 1 [file medinform_v10i1e17278_app1.docx]

*The Use of Blockchain Technology in the Healthcare Sector: a Systematic Review*

*http://dx.doi.org/10.2196/17278*

**Multimedia Appendix 1.** Databases and search terms used.

| Database | Keywords / Search Term |
| --- | --- |
| PubMed | #1 (blockchain OR "block chain")  #2 (healthcare OR health OR medic* OR  medical OR medicine OR *health*)  #3 (usability)  In some cases where the * symbol could not be used, we replaced the original search query with the following:  (blockchain OR "block chain") AND  (healthcare OR health OR medical OR  medicine OR m-health OR mhealth OR  ehealth OR e-health OR telehealth) |
|  | #1 AND #2 AND #3  Date: All article published from Inception to September 2019 |
| Springerlink | #1 (blockchain OR "block chain")  #2 (healthcare OR health OR medic* OR  medical OR medicine OR *health*)  #3 (usability) |
|  | 1# AND 2# AND #3  Date: All article published from Inception to September 2019 |
| IEEE Xplore | #1 (blockchain OR "block chain")  #2 (healthcare OR health OR medic* OR  medical OR medicine OR *health*)  #3 (usability) |
|  | 1# AND 2# AND 3#  Date: All article published from Inception to September 2019 |
| EMBASE | #1 (blockchain OR "block chain").mp.  #2 blockchain$.mp.  #3: #1 OR #2  #4 (healthcare OR health OR medic* OR  medical OR medicine OR *health*).mp.  #5: #3 AND # 4  #6 (usability)$.mp. |
|  | #5 AND #6  Date: All article published from Inception to September 2019 |
| SCOPUS | #1 (blockchain* OR "block chain")  #2 (healthcare* OR health* OR medic* OR  Medical* OR medicine* OR health*)  #3 (usability*) |
|  | 1# AND 2# AND 3#  Data: All article published from Inception to September 2019 |
| EBSCOhost | #1 (blockchain OR "block chain")  #2 (healthcare OR health OR medic* OR  medical OR medicine OR *health*)  #3 (usability) |
|  | 1# AND 2# AND 3#  Date: All article published inception to September 2019 |
