# Supplementary material for: The Use of Blockchain Technology in the Health Care Sector: Systematic Review
Source: JMIR Med Inform. 2022 Jan 20;10(1):e17278. doi: 10.2196/17278 (PMC8814929; doi:10.2196/17278)
Supplement: Multimedia Appendix 2 [file medinform_v10i1e17278_app2.docx]

*The Use of Blockchain Technology in the Healthcare Sector: a Systematic Review*

*http://dx.doi.org/10.2196/17278*

**Multimedia Appendix 2.** PRISMA checklist for the systematic review of the use of blockchain technology in the health care sector.

| **Section/topic** | **#** | **Checklist item** | **Reported on page #** |
| --- | --- | --- | --- |
| **TITLE** | | |  |
| Title | 1 | The Use of Blockchain Technology in Healthcare Sector: A systematic review | i |
| **ABSTRACT** | | |  |
| Structured summary | 2 | Blockchain technology is a new Internet of Things (IoT) application of decentralised, distributed ledgers, immutable and cryptographically secure technology. This technology entails a series of transactions list with identical copies shared and retained by different group or parties. One field where blockchain technology has tremendous potential is healthcare, due to a more patient-centric approach to healthcare system and to connect disparate systems and increase the accuracy of electronic healthcare records. The use of blockchain technology in healthcare sector was reviewed. Therefore, the findings could help the scientific community to understand the use aspect of blockchain technology by reviewing the published studies. The results from this study helps to recognise the accessibility as well as the use of blockchain technology in healthcare sector. This study used systematic review methodology. The existing literature related to the use aspect of blockchain technology in healthcare was included. Relevant articles were searched using Pubmed, Springerlink, IEEE Xplore, Embase, Scopus and EBSCOHost. Quality assessment of literature was performed on the selected  22 articles by assessing the trustworthiness, and relevance. From the search, 34 papers from PubMed, 52 papers form Springerlink, 40 papers from IEEE Xplore, 56 papers from Embase, 45 papers from Scopus and 44 papers from EBSCOHost were identified. After full screening, 22 articles were included. Table of evidence were | iv |

| **Section/topic** | **#** | **Checklist item** | | **Reported on page #** |
| --- | --- | --- | --- | --- |
|  |  | | constructed and interpretation of the results of the selected articles were done. The results of scoring for measuring the quality of the publication were obtained and interpreted. Blockchain technology was found to be useful in real healthcare environment mainly on the management of electronic medical records, biomedical research and education, remote patient monitoring, pharmaceutical supply chain, health insurance claims, health data analytics and some other potential area. The main reason for the implementation of blockchain technology in healthcare sector was identified, namely data integrity, access control, data logging, data versioning and non-repudiation.  Keywords: Blockchain, healthcare, usability, data integrity, access control, data logging, health informatics |  |
| **INTRODUCTION** | | | |  |
| Rationale | 3 | This study provides a detailed insight into different Hospital Information System (HIS) that could be applied in private and government hospitals. The significant of this study on blockchain technology in healthcare sector would give benefits for public, healthcare professionals and also researchers. This study is impactful in helping the scientific community in understanding the use aspect of blockchain technology based on finding of completed studies. The results from this study would help to recognise the accessibility as well as the use of blockchain technology in healthcare sector. | | 4 |
| Objectives | 4 | 1. To systematically review study on use of blockchain technology in healthcare 2. To analyse the characteristics of the use study that have implemented on blockchain technology | | 4 |

| **Section/topic** | | **#** | | **Checklist item** | **Reported on page #** |
| --- | --- | --- | --- | --- | --- |
| **METHODS** | | | | |  |
| Protocol and registration | 5 | | The systematic review method followed in this research was described by (Yli-Huumo et al., 2016). The systematic study was chosen as the research methodology due to the goal which was to learn in detail the existing studies related to the use aspect of blockchain technology in healthcare. Ethical approval were not required as data collection was achieved via published literature. These systemic review study was carried out from September 2018 until July 2019. | | 26 |
| Eligibility criteria | 6 | | A specific inclusion and exclusion criteria were used to screen each paper as follow:   1. Inclusion 2. Original research study 3. Only studies in English were included. 4. Publication on Blockchain technology in healthcare sector 5. Publication including sufficient explanation of the research findings. 6. Exclusion 7. Papers without full text availability 8. Papers where English was not the main language 9. Papers that had some other meaning instead of Blockchain used in Healthcare sector 10. Papers that were duplicates 11. Search that are editorial, prefaces, article summaries, summaries of the tutorials, interviews, news, correspondence discussion, comments, reader’s letters, workshops, panels, and poster sessions 12. Publication indicate ideas, magazine publication and discussion papers | | 32 |

| **Section/topic** | | **#** | | **Checklist item** | **Reported on page #** |
| --- | --- | --- | --- | --- | --- |
| Information sources | 7 | | A comprehensive, electronic search was conducted before March 2019 using PubMed, Springerlink, IEEE Xplore, Embase, Scopus and EBSCOHost. | | 28 |
| Search | 8 | | Please see Table 31. Search Strings Used | | 29-30 |
| Study selection | 9 | | Identification, title and abstract screening, full text eligibility assessment, included in systematic review. | | 31-35 |
| Data collection process | 10 | | The literature was collected using PubMed, Springerlink, IEEE Xplore, Embase, Scopus and EBSCOHost databases. | | 35-36 |
| Data items | 11 | | The literature relating to use study of blockchain technology in healthcare sector from the year 2008 until March 2019 | | 35-36 |
| Risk of bias in individual studies | 12 | | It was not performed in this study because a descriptive studies were included. Furthermore, meta-analysis was not performed for this study. | | - |
| Summary measures | 13 | | Descriptive analysis | | 36-37 |
| Synthesis of results | 14 | | Descriptive analysis | | 36-37 |
| Risk of bias across studies | 15 | | Not applicable because meta-analysis was not performed for this study | | - |
| Additional analyses | 16 | | Not applicable because meta-analysis was not performed for this study | | - |

| **Section/topic** | **#** | **Checklist item** | **Reported on page #** |
| --- | --- | --- | --- |
| **RESULTS** | | |  |
| Study selection | 17 | Refer to Figure 4.2 | 38-45 |
| Study characteristics | 18 | Refer to Table 4.3 : Table of Evidence | 51-71 |
| Risk of bias within studies | 19 | Not applicable | - |
| Results of individual studies | 20 | Refer to Table 4.3 : Table of Evidence | 51-71 |
| Synthesis of results | 21 | Presented in Discussion Chapter | 78-89 |
| Risk of bias across studies | 22 | Not applicable | - |
| Additional analysis | 23 | Not applicable | - |

| **Section/topic** | **#** | **Checklist item** | **Reported on page #** |
| --- | --- | --- | --- |
| **DISCUSSION** | | |  |
| Summary of evidence | 24 | The result from this systematic review study show that the majority of the research regarding the blockchain technology in healthcare environment was focused on the management of electronic medical records followed by biomedical research and education, remote patient monitoring, pharmaceutical supply chain, health insurance claims, health data analytics and some other potential area. About 10 (45%) of the selected paper concentrates on the management of electronic medical records. The application of blockchain in management of electronic health record will make sure the data sharing become easier, transparent and trustworthy among the healthcare stakeholder and also the patient may have the control over their own data (Cunningham & Ainsworth, 2017).  About 23% of the selected paper in this study indicate that the blockchain could be applied in biomedical research and education fields. Blockchain technology had been used extensively in biomedical research and education to preserve the data privacy, integrity, sharing, record sharing, record keeping especially in clinical trials (Nugent et al., 2016). In clinical trials, according to (Angeletti et al., 2017), blockchain can aid to abolish falsification of data and under-reporting of unwanted results of the clinical research. Remote patient monitoring was another blockchain use case in healthcare sector. In mobile healthcare environment, (Liang et al., 2017) presents a Hyperledger-based implementation of blockchain-enabled data collection and sharing between the healthcare stakeholders. Drug or pharmaceutical supply chain is one of the use cases of blockchain technology in healthcare sector, particularly health supply chain management. Blockchain had been applied in this field to allocate safe and secure platform and also to address the most common problems faced in pharmaceutical industry such as delivery of substandard or counterfeit medication which may lead to greater negative impact to the patient. | 78-89 |

| **Section/topic** | **#** | **Checklist item** | **Reported on page #** |
| --- | --- | --- | --- |
|  |  | Health Insurance is necessary in everyone life which guarded an individual property from unbelievable cost of a medical treatment. The medical insurance data of a patient can be encrypted and immutably stored on blockchain which enhanced credibility and eliminated the involvement of third party in the management of the health insurance of a patient (Zhou et al., 2018). Blockchain in collaboration with other emerging technologies such as deep and transfer learning techniques were used to perceive predictive analytics of healthcare data. (Kotsiuba et al., 2018) stated that blockchain provide a unique opportunity to overcome the problems related with analysis and security of medical data. A study by (Talukder et al., 2018) presented a blockchain consensus protocol which provides an accurate medical decisions and reducing the disease burden by using Ethereum based on proof of disease consensus prorocol The main reason for the application or implementation of blockchain technology in healthcare sector was identified and categorised into data integrity (41%), followed by access control (32%), data logging (18%), data versioning (6%) and non-repudiation from the selected papers in this study.  Data integrity is defined as the accuracy and consistency of the data or information stored in the system which act as an important component of information security. Data integrity was achieved by using the blockchain technology in healthcare sector. Firstly, (Li et al., 2018) implemented blockchain based platform Ethereum to maintain the originality and variability of stored data in the system while preserving privacy for user. The maintenance of data in blockchain for lifelong was achieved with the concept of proof of primitiveness of data and the system can validate the data where it is identical to the original data. The data could be restored and verified through blockchain if the data had been damages. |  |

| **Section/topic** | **#** | **Checklist item** | **Reported on page #** |
| --- | --- | --- | --- |
|  |  | According to (Cunningham & Ainsworth, 2017), access control are defined as an individual that has the full authorities in deciding who, when and how much of their own medical data can be accessed using the blockchain technology. Access control may lead to direct patient involvement in controlling their own medical data usage. The distribute ledger which acts as one of the characteristics of blockchain technology ensure efficient access and retrieval of electronic medical records (Fan et al., 2018).  Data logging is defined as an operation of gathering and storing information over a period of time. It allows tracking of all type of interaction such as storage, accessing or modification of data, files or application in a system. Data logging can be achieved by the application of blockchain technology in healthcare sector. In clinical trials, (Nugent et al., 2016) demonstrated the blockchain technology used an Ethereum smart contract to enhance the trust, reliability and transparency of data management. The cryptographic and tamperproof characteristics of blockchain avoid all forms of manipulation and enhance the data logging of complex clinical trial data management, where a better informed decision can be made by the medical professional.  Data versioning is defined as saving a new copies of the data when any modification was made on the existing data. This helps to keep tracking the data and also ensure easy retrieval of any specific version of the respective stored data in a system. (Kleinaki et al., 2018) implemented a blockchain based notarization services which uses smart digital contracts to secure the data in biomedical research sector. |  |

| **Section/topic** | **#** | **Checklist item** | **Reported on page #** |
| --- | --- | --- | --- |
|  |  | Non-repudiation guarantees the validity of data in a particular healthcare system which could not be denied by anyone and ensures the originality and integrity of data. Study by (Benchoufi et al., 2017) used the blockchain technology to collect, store and track the clinical trial consent in a secure, unfalsifiable and publically verifiable way which are originally time-stamped with the application of proof of concept lead to non-repudiation of data. The authentication system ensure the clinical trial consent are accessible and transparent for patient while traceable for stakeholders. |  |
| Limitations | 25 | The limitation of this systematic review study was there was no published study on the safety of blockchain technology in healthcare, therefore the safety aspect of the blockchain technology cannot be reviewed. There were almost no or fewer paper published the negative aspects of implementation of blockchain technology in healthcare. Most studies only published the positive aspect of the blockchain technology in healthcare which may lead to biasness. | 90 |
| Conclusions | 26 | Blockchain technology is still a fairly new technology that has not been widely implemented in healthcare sector. This study can be a guide for a future research, implementation and evaluation of blockchain technology in healthcare sector. More research should be carried out regarding the implementation of blockchain technology in real healthcare environment for a better understanding, characterization and evaluation of blockchain technology in healthcare. Additional focus should be given by researchers to carry out researches regarding safety on implementation of blockchain technology in healthcare. | 90 |
| **FUNDING** | | |  |
| Funding | 27 | This systematic review received no funding | - |
